# An automated classification pipeline for tables in pharmacokinetic literature

**DOI:** 10.1038/s41598-025-94778-5

**Published:** 2025-03-24

**Authors:** Victoria C. Smith, Ferran Gonzalez Hernandez, Thanaporn Wattanakul, Palang Chotsiri, José Antonio Cordero, Maria Rosa Ballester, Màrius Duran, Olga Fanlo Escudero, Watjana Lilaonitkul, Joseph F. Standing, Frank Kloprogge

**Affiliations:** 1 Institute of Health Informatics, University College London, London, UK; 2 Great Ormond Street Institute for Child Health, University College London, London, UK; 3 Department of Computer Science, University College London, London, UK; 4 Mahidol Oxford Tropical Medicine Research Unit, Faculty of Tropical Medicine, Mahidol University, Bangkok, Thailand; 5 Clinical Pharmacology, Modelling and Simulation, Parexel International, Bangkok, Thailand; 6 Blanquerna School of Health Sciences, Ramon Llull University, Barcelona, Spain; 7 Institut de Recerca Sant Pau Barcelona, Barcelona, Spain; 8 Global Business School for Health, University College London, London, UK; 9 Department of Pharmacy, Great Ormond Street Hospital for Children, London, UK; 10 Institute for Global Health, University College London, London, UK

**Keywords:** Literature mining, Machine learning, Clinical pharmacology, Pharmacokinetics

## Abstract

Pharmacokinetic (PK) models are essential for optimising drug candidate selection and dosing regimens in drug development. Preclinical and population PK models benefit from integrating prior knowledge from existing compounds. While tables in scientific literature contain comprehensive prior PK data and critical contextual information, the lack of automated extraction tools forces researchers to manually curate datasets, limiting efficiency and scalability. This study addresses this gap by focusing on the crucial first step of PK table mining: automatically identifying tables containing in vivo PK parameters and study population characteristics. To this end, an expert-annotated corpus of 2640 tables from PK literature was developed and used to train a supervised classification pipeline. The pipeline integrates diverse table features and representations, with GPT-4 refining predictions in uncertain cases. The resulting model achieved F1 scores exceeding 96% across all classes. The pipeline was applied to PK papers from PubMed Central Open-Access, with results integrated into the PK paper search tool at www.pkpdai.com. This work establishes a foundational step towards automating PK table data extraction and streamlining dataset curation. The corpus and code are openly available.

## Introduction

Pharmacokinetics (PK) plays a crucial role in drug development by quantifying drug exposure through the processes of absorption, distribution, metabolism, and excretion (ADME). The selection of drug candidates and the determination of therapeutically relevant doses and dosing schedules rely on the adequate characterisation of PK profiles^1,2^. Mathematical modelling of PK processes has become increasingly important for optimising drug candidate selection and dosing regimens throughout the drug development process.

Prior PK data from similar compounds are valuable for first-in-man prediction of novel compounds and initialising population PK models^3,4^. However, in vivo PK parameter data are typically reported in free-text and semi-structured tables within the scientific literature, making them challenging to access automatically. Existing PK databases manually compile PK data, covering only a limited subset of drugs^5–7^. As a result, PK researchers must often engage in labour-intensive manual searching and extracting of relevant PK data, a challenge exacerbated by the growing volume of published research.

Automating the extraction of PK data from tables presents the opportunity to streamline dataset curation and improve PK parameter predictions. Existing automated tools for PK information extraction focus on free-text data^8–10^. However, free-text descriptions often fragment key information, separating PK values from essential contextual details such as study conditions and patient characteristics. In contrast, tables consolidate comprehensive PK data alongside relevant metadata in a semi-structured format, making them a highly valuable yet underutilised resource. Despite this, no prior work has addressed automated PK data extraction from tables, leaving a critical gap in leveraging this rich source of information.

Table mining, the automated retrieval of information from tables, has been studied across various domains^11–15^. Broadly, previous works frame table-mining as the following subtasks: (i) table detection, identifying the location and boundaries of tables in documents; (ii) table type classification, determining whether a table contains relevant data for extraction; (iii) entity recognition, identifying specific entities of interest in table cells (such as PK parameters, units, or patient characteristics); and (iv) linking extracted results to structured knowledge bases. While table detection (step i) is mainly domain-independent, steps (ii) through (iv) require domain-specific tailoring, particularly in biomedical literature, due to specialised terminology and diverse tabular formats^13^. PK tables necessitate a tailored table mining approach due to the variable notation of PK parameters, diverse table structures across studies, and the need to contextualise PK estimates with patient characteristics.

In the PK domain, an automated data extraction pipeline for tables would enable the efficient curation of a comprehensive and centralised PK database, improving access to valuable data for modelling and research. However, no previous work has addressed table mining for PK literature. A significant challenge is distinguishing relevant PK data tables from ones containing ancillary information such as in vitro chemical data, stability test results, concentration measurements without derived PK estimates, pharmacodynamic endpoints, clinical endpoints, and adverse events. Without an initial classification step, automated pipelines must process vast amounts of irrelevant tables, leading to unnecessary computational overhead, reduced extraction accuracy, and increased noise in curated datasets. By pre-filtering relevant tables, classification enhances efficiency and ensures that subsequent extraction focuses on high-value data. To address this, this study focuses on table classification, the first domain-dependent step of table mining, to determine relevant tables containing in vivo PK parameter estimates or patient characteristics. This crucial filtering step ensures only relevant tables proceed to subsequent extraction steps, enhancing the accuracy, efficiency, and scalability of large-scale PK data extraction.

While domain experts can readily identify relevant tables in PK literature, automating this process presents significant challenges. These challenges include the heterogeneous structures and layouts of tables, the variability in information placement across table captions, bodies, and footers, the diversity of PK parameter surface forms, and the dilution of relevant information within large volumes of table text. Such variability makes heuristic approaches insufficient, necessitating a more flexible and generalisable solution. Machine learning (ML) approaches, which can learn complex patterns, offer a scalable and flexible solution.

Training effective ML models require high-quality annotated data. However, no such dataset currently exists for PK table classification. This study addresses this need by introducing a new expert-annotated corpus of tables containing PK parameter estimates and study population characteristics. Additionally, it presents a classification pipeline trained and evaluated on this corpus. Primarily, this pipeline lays the foundations for large-scale PK data extraction from tables in scientific literature. Additionally, PK researchers can use this classification pipeline independently as a search aid to efficiently identify relevant tables to review in detail.

The key contributions of this study are as follows:

- The PK Table Classification (PKTC) corpus, comprising 2640 manually annotated tables from scientific literature, labelled for the presence of PK parameter data and study population characteristics.
- A supervised machine-learning-based classification pipeline, trained and evaluated on the PKTC corpus, with an assessment of the impact of domain-specific pretraining.
- A hybrid approach integrating GPT-4o to refine classification predictions in cases of uncertainty.
- Large-scale application of the classification pipeline to PK literature from PubMed Central Open-Access, with the integration of results into the PK paper search tool from Hernandez et al.^9^ at www.pkpdai.com.

## Methodology

### Corpus development

The PKTC Corpus was developed to train and evaluate pipelines to classify whether tables contain PK parameter estimates. This can be found at https://zenodo.org/records/13884895.

#### Data source

A multi-step pipeline was employed to create a candidate pool of tables. Publications reporting novel PK parameters from in vivo studies were initially identified through PubMed using the PK document search tool developed by Hernandez et al.^9^, which includes publications up to and including 2021. A total of 10,132 articles with full-text access from the PMC Open Access subset (https://www.ncbi.nlm.nih.gov/pmc/tools/openftlist/) were downloaded in XML format from PMC (https://ftp.ncbi.nlm.nih.gov/pub/pmc/). Articles only available as abstracts were excluded from further processing. Tables were extracted from the full-text XML papers by targeting the content within specific HTML table tags (e.g., captions, rows, footers) associated with the table structure. Tables available only as images in the papers were excluded from the pool. This process resulted in a final pool of 12,030 tables. From this pool, a randomised selection of 2640 tables was chosen for annotation. The dataset was split into training, validation, and test sets, containing 1584, 528, and 522 samples, respectively.

#### Annotation

**Protocol** The annotation team consisted of seven pharmacometricians with substantial expertise in PK and familiarity with study types prevalent in PK literature. To ensure consistent annotation, detailed guidelines were provided to all annotators (see Appendix A.2). Initially, each table in the training, validation, and test sets was independently annotated by two annotators. In cases of conflicting annotations, review sessions were conducted after each iteration to resolve disagreements. The guidelines were updated iteratively to capture these resolutions and inform future annotation decisions. Cohen’s Kappa Coefficient (see Eq. 1) was calculated before conflict resolution to assess inter-annotator agreement.

1 $$\begin{aligned} K = \frac{(p_{o} - p_{e})}{(1 - p_{e})} \end{aligned}$$

where $p_o$ is the observed agreement, and $p_e$ is the probability of agreement by chance.

**Interface** A custom labelling interface was developed using the commercial tool Prodigy^16^. An example can be seen in Appendix A.1. The interface displayed the full table, caption, and footer, and domain experts were asked to determine whether the table contained PK parameter data. If annotators were uncertain about labelling a specific table, they could flag it for review and leave comments for further clarification.

**Guidelines** Detailed annotation guidelines were provided to the annotation team before the task to promote consistency in decision-making. The guidelines were updated after each annotation round to address challenges and ambiguities encountered. Uncertainties were discussed within the team, and decisions were made based on the intended application of the classifier. The final version of the annotation guidelines can be seen in the Appendix A.2.

### Classifcation pipeline development

Due to the heterogeneity of tables found in the PK literature, a machine learning-based approach was developed to identify tables that present PK parameter data.

#### Field selection

Tables in the PKTC dataset can be extensive, with some exceeding 6000 tokens, and relevant information is often located deep within the table. However, passing large volumes of redundant text to the classifier introduces noise, potentially reducing classification accuracy. A series of preprocessing variations were applied to explore the trade-off between including all relevant information and minimising noise. The following table fields were extracted and evaluated for their impact on classification performance:

1. Caption
2. Header row (markdown format)
3. First column (concatenated)
4. First few rows (markdown format)
5. Table (markdown format)
6. Footer

The tables were converted to markdown format, serialising the content row by row. This format was chosen for its simplicity and closer resemblance to natural language.

#### Representations

A simple bag-of-words (BoW) model was compared to various distributed representations for encoding tables. BoW transforms text into a fixed-length vector by counting the frequency of each word in a predefined vocabulary. In this approach, each unique word corresponds to a feature in the vector, with its value representing how often the word appears in the document. Although straightforward, BoW fails to capture contextual or semantic meaning.

In contrast, distributed representations encode contextual and semantic information into dense, fixed-size vectors within a predefined vector space. These representations have lower dimensionality than BoW and are learned through model pre-training on large corpora using self-supervised tasks (e.g., masked language modelling). Several pre-trained transformer-encoder models were tested to generate caption and table representations, each with different pre-training focuses, including general text, biomedical text, and text embedding tasks. The models evaluated include:

1. Bidirectional Encoder Representations from Transformers (BERT)^17^: Pretrained on a large corpus of unstructured text (BooksCorpus and English Wikipedia) using self-supervised objectives like masked language modelling and next sentence prediction. BERT learns word correlations, enabling it to generate context-dependent vector representations.
2. BioBERT^18^: A version of BERT further pretrained on biomedical text, including PubMed abstracts and full-text articles from PubMed Central. This additional training makes BioBERT more suitable for biomedical applications.
3. PubMedBERT (BiomedBERT)^19^: Based on the BERT architecture, pretrained specifically on PubMed abstracts and full-text articles, PubMedBERT is optimised for biomedical text representation.
4. all-mpnet-base-v2^20^: Based on the MPNet transformer architecture, pretrained on general text corpora such as BooksCorpus and English Wikipedia, using masked and permutation-based learning tasks. Fine-tuned for text embedding, it captures contextual relationships between sentences and positions similar texts close together in the embedding space.
5. E5-small-v2^21^: Part of the E5 model family, pretrained on diverse web-based corpora (e.g., MS MARCO, Natural Questions) using a contrastive learning objective. This enables the model to learn relationships between text pairs, making it well-suited for downstream tasks like classification.

**Dealing with long texts** The distributed representation models used in this study have a maximum input length of 512 tokens, yet approximately 25% of tables in the dataset exceed this limit. To address this, tables were divided into 512-token chunks, each encoded separately. These chunk representations were then pooled to form a single vector representing the table. Two pooling methods were compared: (1) mean-pooling, which calculates the average of all feature values across the chunk vectors, and (2) max-pooling, which selects the maximum value for each feature, emphasising the most significant information from the chunk vectors.

#### Zero-shot classification with generative LLMs

Large pretrained language models (LLMs), such as Generative Pretrained Transformers (GPT), exhibit emergent abilities as they scale through extensive pretraining on diverse text corpora^22^. One such ability is zero-shot learning, where the model can classify data it has not encountered during training, relying solely on label descriptions and inclusion/exclusion criteria provided through a prompt. Chain-of-thought (CoT) prompting, which involves breaking down a problem into sequential logical steps, has been shown to enhance the performance of LLMs in solving complex tasks^23^.

GPT-4o was selected to classify the PKTC corpus without task-specific training in this experiment due to its superior zero-shot learning capabilities, which arise from its extensive pretraining on diverse data sources. The annotation guidelines, improved across iterative annotation rounds, were used to develop a comprehensive CoT prompt (see Appendix B.1), leveraging GPT-4o’s reasoning capabilities to improve the model’s accuracy by guiding it through a series of logical steps.

### Combining supervised methods and zero-shot methods

Given the goal of applying this classification pipeline to all tables from the PubMed Open Access (full-text) corpus and reusing the approach as there are new publications, a hybrid of the two approaches was proposed to balance inference speed and costs. Here, the LLM is used for zero-shot classification only in cases where the supervised classifier’s confidence is below a certain threshold. This method provides a “double-check” for uncertain instances only. The threshold was determined by plotting the percentage of the dataset and the F1-score on this percentage at different confidence thresholds on the validation set (see Appendix C). The threshold was selected to maximise the number of incorrect predictions under this whilst balancing this with the dataset percentage.

#### Classifier

Extreme Gradient Boosting (XGBoost) is a scalable, efficient implementation of the gradient boosting framework^24^. XGBoost has demonstrated superior performance in various machine learning tasks, including text classification^24–26^.

XGBoost constructs an ensemble of weak learners, typically decision trees, to build a robust predictive model. In each iteration, new trees are trained to correct errors from previous ones, optimising an objective function that balances model fit with regularisation to prevent overfitting. The general objective function is expressed as:

2 $$\begin{aligned} F(x) = \sum _{m=1}^M\gamma _{m}h_{m}(x) \end{aligned}$$

,where *M* is the total number of boosting iterations, $\gamma _m$ is the weight applied to the *m*-th weak learner, and $h_m (x)$ represents the weak learner’s contribution. New learners are added iteratively until the maximum number of iterations is reached.

Several factors motivated the choice of XGBoost for PK table classification. Unlike linear models such as logistic regression and SVM, XGBoost captures nonlinear relationships and complex feature interactions, making it well-suited for table-text data. Its tree-based structure efficiently handles sparse, high-dimensional feature spaces typical of BoW representations. Built-in regularisation and gradient-based optimisation enhance robustness against noise, while weighted loss functions and boosting iterations mitigate class imbalance - critical for the PKTC dataset. Compared to deep learning models, which demand extensive labelled data and high computational resources, XGBoost delivers competitive performance with greater efficiency and scalability for large-scale applications.

XGBoost was trained to minimise cross-entropy loss, with sample weights adjusted inversely to class frequencies in the PKTC corpus to mitigate class imbalance.

**Hyperparameter tuning**

Hyperparameter tuning was performed using a 10-fold cross-validation grid search. This approach helps estimate model generalisability and reduces bias and variance. The training data was shuffled and split into ten folds, with the model trained on nine folds and tested on the remaining fold. This process was repeated ten times, and the final performance metrics were averaged.

For hyperparameter selection, candidate values were specified (Table 1), while the learning rate was fixed at 0.1. Early stopping was applied to prevent overfitting, halting the boosting process after ten rounds without improvement in AUC-ROC. The number of boosting rounds was capped at 1000, while other parameters were set to scikit-learn defaults.

**Table 1:** Hyperparamters, tuned over which ranges.

Hyperparameter	Description	Range (step size)
Max depth	Maximum tree depth	2–10 (2)
Min child weight	Minimum sum of instance weight (hessian) needed in a child node	1–6 (2)
Gamma	Minimum loss reduction required to make a split	0.1–0.5 (0.1)
Subsample	Fraction of training data used to grow each tree	0.5–1.0 (0.1)
Colsample bytree	Fraction of features used to build each tree	0.3–1.0 (0.1)

### Evaluation

The supervised pipeline development aimed to optimise classifier performance across all classes by exploring different configurations and tuning hyperparameters. Performance was evaluated based on variations in (1) input table fields, (2) table representations, and (3) pipeline hyperparameters. Precision, recall, and the $F_{1}$ score (the harmonic mean of precision and recall) were used to compare pipeline variants. For multi-class classification, micro-averages (global averages, not distinguishing between classes) and macro-averages (taking the mean across the metric for each class, treating each class equally) for each metric were calculated.

Hyperparameter tuning was performed using 10-fold cross-validation to provide a robust performance estimate on the combined training and validation sets. The final best classifier pipeline was trained on the entire training and validation datasets with optimised hyperparameters and evaluated on the test set to measure performance on unseen data.

For the zero-shot analyses, the prompt was developed using examples from the training set and applied directly to the test set to generate performance metrics.

## Results and discussion

### Annotator agreement and label statistics

To assess annotation quality and consistency, we calculated the inter-annotator agreement using Cohen’s Kappa score (*K*) before splitting the dataset. The initial agreement score, calculated pairwise between annotators on the first 100 tables annotated, was $K=0.78\pm 0.03$. The primary sources of disagreement were edge cases, where the distinction between PK and non-PK tables was ambiguous, specifically (1) Tables containing individual and mean drug concentration measurements and (2) PK parameters included in Pharmacodynamic endpoints, (3) tables containing mixed content, making relevant data hard to detect initially, (4) tables using non-standard terminology. We improved the annotation process through team discussions and guideline refinements until we achieved pairwise $K = 0.9$ on the full dataset.

Following agreement on all annotations, the dataset was divided into training (60%), development (20%), and test (20%) sets. The proportions of each class were maintained across splits to ensure representative sampling. Table 2 shows the distribution of labels across these splits and the total counts for each category in the complete dataset. The distribution of labels reflects the real-world prevalence of different table types in PK literature, with ’Other’ being the most common category. This imbalance was addressed during model training.

**Table 2:** Summary of the PKTC corpus statistics.

Dataset	# PK	# Demographics	# Other	# Total
Training	532	233	819	1584
Development	178	78	273	529
Test	177	77	273	527
**Total**	887	388	1365	2640

The dataset was split into training (60%), development (20%), and test (20%) sets after completing the annotation process and resolving inter-annotator disagreements.

### Table field selection

Firstly, different features from the tables were represented as BoW features and used to train the XGBoost classifier. The results of this analysis are presented in Table 3. Notably, using features derived from the table footer, header row, or first column yielded significantly poorer performance than the caption or the whole table. The full table gave the highest results of a single feature, highlighting the importance of information spanning the entire table for the classification task.

The caption and the table emerged as the top performers among the evaluated features. These two features were concatenated (caption + table), achieving the best results overall, indicating that these combined elements provide a more comprehensive context for classification. Our findings align with those of Kardas et al.^15^, who classified machine learning results tables based on their captions using a classifier built on the ULMFiT architecture, pretrained on text from arXiv papers. They reported a macro F1-score of 64% for their two primary classes. While their approach highlights the potential of captions in table classification, our results suggest that incorporating the table alongside the caption can yield even greater classification accuracy.

**Table 3:** Summary performance metrics of different features used in the table field selection analysis.

Table field	$P_{macro}$	$R_{macro}$	$F1_{macro}$	$F1_{micro}$
Caption	$92.04 \,(1.66)$	$92.3 \,(1.95)$	$92.09 \,(1.69)$	$91.42 \,(1.59)$
Header row	$71.27 \,(4.03)$	$70.21 \,(4.45)$	$70.49 \,(4.21)$	$74.24 \,(3.16)$
First column	$87.06 \,(1.7)$	$85.52 \,(2.46)$	$85.87 \,(2.02)$	$85.86 \,(1.92)$
First few rows	$89.49 \,(2.35)$	$90.01 \,(1.96)$	$89.62 \,(2.04)$	$89.65 \,(1.63)$
Table	$92.3 \,(1.94)$	$93.15 \,(1.98)$	$92.53 \,(1.89)$	$92.42 \,(1.93)$
Footer	$74.3 \,(4.32)$	$68.77 \,(4.92)$	$70.55 \,(4.83)$	$74.5 \,(3.68)$
Caption + table	$94.97 \,(1.7)$	$94.71 \,(1.95)$	$94.77 \,(1.8)$	$94.32 \,(1.73)$

Results are $mean \,(s.d.)$ from 10-fold cross-validation on the training set. All runs were performed using BoW features and an XGBoost classifier.

### Representation approaches

Several representation methods were compared, including BoW and distributed representations. A key challenge observed was that the Caption + Table feature length often exceeds the maximum input token length for transformer-encoder models (see Appendix B.2). Specifically, 25% of the examples in the corpus exceeded this limit, leading to truncation during a single pass through the language model, which could lead to potential information loss. To address this, longer texts were chunked for distributed representations, and each chunk was embedded separately using a pre-trained language model, followed by either mean or max pooling over these chunks.

**Fig. 1:**
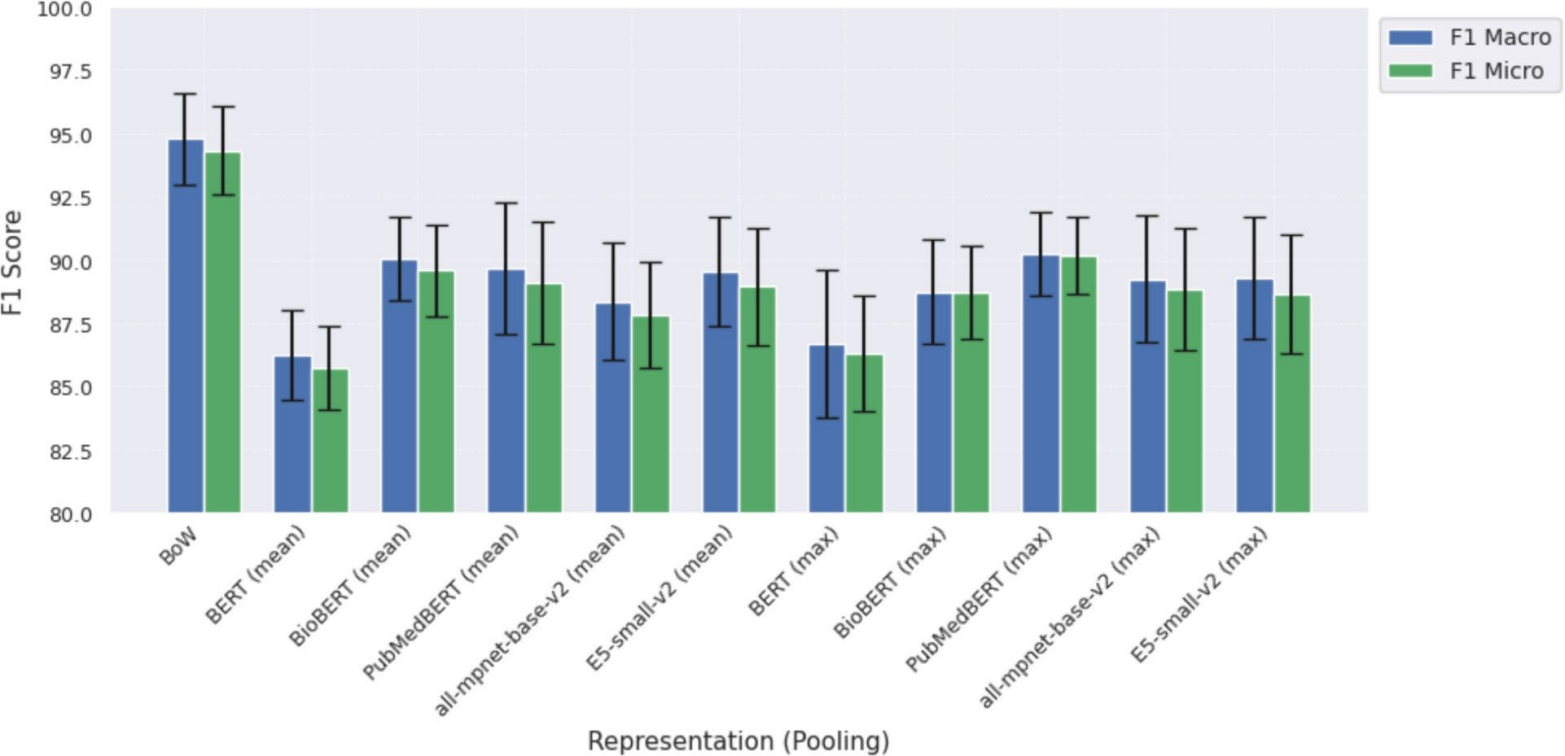
Macro- and micro-F1 scores for different representation methods. Results are shown as mean with error bars as standard deviations from 10-fold cross-validation on the combined training set. All runs were performed with an XGBoost classifier.

The performance of different representations is summarised in Fig. 1 and detailed in Table 4. Interestingly, the simplest method, BoW, outperformed pre-trained language model representations. This suggests that simple word frequencies effectively distinguish between classes for this task. At the same time, deeper contextual relationships captured by transformer-based models may introduce noise rather than functional features. This could be because none of the pre-trained models used were specifically fine-tuned for tabular data, so their embeddings might not align well with the nuances of the task at hand. However, Koleva et al.^27^ found table-specific pre-trained models underperformed compared to BERT^17^ on a table classification task.

Among the distributed representations, BERT performs the worst across the board. The general-domain models E5 and all-mpnet-base-v2 perform slightly less than biomedical domain-specific models for mean pooling. However, they do marginally better than BioBERT but not PubMedBERT when using max-pooling. This indicates that task-relevant (e.g., embedding) training provides benefits similar to domain specialisation. Our findings contrast with those of Koleva et al.^27^, who explored different representations for web table classification using a Multi-layer Perceptron (MLP). They compared TF-IDF, pretrained word vectors, and transformer-based models such as BERT and TaBERT. Notably, in their study, BERT achieved the best performance (macro-F1 score of 0.80), while TF-IDF performed the worst. Their dataset, with fewer than 300 tables and a much greater number of classes, likely benefited more from context-rich BERT embeddings, which can help in low-resource scenarios. Additionally, they used sampling the first few rows of tables to manage scalability issues, which may have contributed to the discrepancy.

**Table 4:** Summary performance metrics of different representation methods.

Representation	Pooling	$P_{macro}$	$R_{macro}$	$F1_{macro}$	$F1_{micro}$
BoW	-	$\mathbf {94.97} \,(\mathbf {1.7})$	$\mathbf {94.71} \,(\mathbf {1.95})$	$\mathbf {94.77} \,(\mathbf {1.8})$	$\mathbf {94.32} \,(\mathbf {1.73})$
BERT	Mean	$87.35 \,(2.48)$	$85.44 \,(1.71)$	$86.23 \,(1.79)$	$85.73 \,(1.64)$
BioBERT	Mean	$91.08 \,(1.95)$	$89.33 \,(1.94)$	$90.05 \,(1.63)$	$89.58 \,(1.81)$
PubMedBERT	Mean	$90.35 \,(2.66)$	$89.12 \,(2.8)$	$89.66 \,(2.62)$	$89.07 \,(2.4)$
all-mpnet-base-v2	Mean	$89.59 \,(1.86)$	$87.45 \,(3.01)$	$88.35 \,(2.3)$	$87.82 \,(2.08)$
E5-small-v2	Mean	$90.88 \,(2.27)$	$88.48 \,(2.31)$	$89.54 \,(2.18)$	$88.95 \,(2.32)$
BERT	Max	$89.0 \,(2.76)$	$85.11 \,(3.23)$	$86.68 \,(2.89)$	$86.3 \,(2.29)$
BioBERT	Max	$90.81 \,(2.06)$	$87.28 \,(2.68)$	$88.72 \,(2.07)$	$88.7 \,(1.84)$
PubMedBERT	Max	$91.95 \,(1.1)$	$88.91 \,(2.28)$	$90.23 \,(1.67)$	$90.15 \,(1.53)$
all-mpnet-base-v2	Max	$90.71 \,(2.3)$	$88.09 \,(2.93)$	$89.24 \,(2.51)$	$88.83 \,(2.42)$
E5-small-v2	Max	$90.77 \,(2.62)$	$88.17 \,(2.44)$	$89.3 \,(2.42)$	$88.64 \,(2.34)$

Results are $mean \,(s.d.)$ from 10-fold cross-validation on the training set. All runs were performed with an XGBoost classifier.

### Zero-shot classification and combining approaches

After tuning the final XGBoost classifier using BoW features, we applied the model to the test set to evaluate its performance on unseen data. The optimised hyperparameter values are detailed in Appendix B.3. The classifier demonstrated over 94% F1 score across all three classes (Macro-F1 of 95.79) in the unseen test set. The results of this analysis are presented in Table 5.

XGBoost was chosen as the classification algorithm because it can efficiently handle high-dimensional, sparse feature spaces while maintaining strong generalisation through built-in regularisation. The results indicate that XGBoost has efficiently captured key discriminative patterns in the table data, enabling robust classification of PK tables despite the noisy and sparse input data. XGBoost’s gradient-based optimisation and weighting of the loss function also helped to mitigate class imbalance, contributing to consistently high F1 scores across all classes.

To explore the capabilities of advanced models, we compared the XGBoost classifier’s performance with a zero-shot approach utilising a chain-of-thought prompt directed at GPT-4o (which features a 128K token context window). The zero-shot method performs comparably with the XGBoost classifier, achieving a Macro-F1 of 95.57. Hegselmann et al.^28^ employ a zero-shot approach using LLMs to answer binary questions related to serialized input tables and, similarly, they observed that a zero-shot methodology even outperforms pretrained neural models and tree-based classifiers.

While the zero-shot approach performed comparably to the supervised method, our pipeline is designed for large-scale literature mining, where efficiency is critical. XGBoost provides a scalable and computationally efficient solution, but relying on it alone risks misclassifications in ambiguous cases. To balance efficiency and accuracy, we implemented a hybrid approach that strategically integrates GPT-4o’s reasoning capabilities. Specifically, when the XGBoost classifier’s confidence fell below a predefined threshold, GPT-4o performed zero-shot classification, effectively serving as a “double check” for uncertain cases. We evaluated three threshold values, with $T=0.9$ yielding the highest overall performance across all classes. This hybrid strategy improved classification accuracy across all tested thresholds while minimising GPT-4o usage, with fewer than 10% of cases requiring double-checking. By leveraging the strengths of both methods, our approach achieves superior scalability and performance.

**Table 5:** Results of the best-supervised classifier, compared with zero-shot GPT-4o and a combined approach on the test set.

Approach	PK			Demographics			Other			Macro F1
	*P*	*R*	*F*1	*P*	*R*	*F*1	*P*	*R*	*F*1	
Best classifier	95.91	92.66	94.25	96.25	98.72	97.47	94.95	96.34	95.64	95.79
Zero-shot GPT-4o	98.72	92.77	95.65	97.07	94.98	96.01	92.09	98.19	95.04	95.57
Combined ($T=0.85$)	98.22	93.79	95.95	97.47	98.72	98.09	96.07	98.53	97.29	97.11
Combined ($T=0.9$)	98.82	94.35	96.53	96.25	98.72	97.47	96.42	98.53	97.46	97.15
Combined ($T=0.95$)	98.82	94.35	96.53	95.06	98.72	96.86	96.40	98.17	97.28	96.89

*T* is the confidence threshold. Results are shown as precision, recall and F1 score per class.

### Large-scale application

Finally, we applied our pipeline to the PK papers from PubMed Open Access. We incorporated the results into the PKPDAI paper search tool from Hernandez et al.^9^(at www.pkpdai.com). This involved several steps. Firstly, as the PKPDAI paper search has not been updated since September 2021, we initially ran the PK paper classification pipeline^9^ across all papers under the search “Pharmacokinetic” published between September 2021 and October 2024 (finding 47,073 papers). We updated the PKPDAI tool with these up-to-date results. We identified 14,100 of these PK papers available as full-text XMLs from PubMed Open Access. We downloaded and parsed these papers to extract 32,862 tables. The table classification pipeline was then applied to identify 10,496 tables containing in vivo PK parameter data and 3627 tables containing demographic data. The confidence threshold for the combined method selected 3183 tables for “double check”. Following this, 100 tables were randomly selected and annotated, and performance metrics were calculated on this subset (see Table 6) to observe the pipeline robustness to real-world data. Finally, the online paper search was updated, adding functionality to enable filtering by open-access papers and specifying the location of tables containing PK parameters and demographic information within these papers.

**Table 6:** Results of the best pipeline on 100 manually inspected examples from the large-scale application.

PK			Demographics			Other			Macro F1
*P*	*R*	*F*1	*P*	*R*	*F*1	*P*	*R*	*F*1	
90.91	85.71	88.24	100.00	90.00	94.74	89.66	94.55	92.04	91.67

### Limitations

While our pipeline effectively identifies relevant tables, several significant limitations must be considered. The current scope of the table classification pipeline is restricted to open-access PMC papers available for text mining (in full-text), excluding potentially valuable data in subscription-based journals. Additionally, tables available only as images are not processed, further limiting coverage. These constraints mean the tool cannot capture all relevant PK data, particularly from older literature and specialised journals.

The development of this pipeline represents a crucial first step in automating PK information extraction. However, as a standalone, expert review of identified PK data remains necessary to evaluate their relevance, quality and context. These limitations make the standalone tool effective only as an initial screening step to help identify PK data for expert analysis. Future work is required, focusing on automating the extraction of parameter values, units, and associated patient characteristic information to extract PK data with the broader context needed to interpret it.

## Conclusion

This study introduces a PK table classification pipeline to identify tables reporting in vivo PK parameters and patient characteristic information, representing a crucial first step towards automating PK data extraction from tables in scientific literature. To support this effort, a novel expert-annotated dataset of tables from PK literature was developed. A hybrid classification approach was trained on this dataset, integrating a robust XGBoost classifier with GPT-4 to improve the handling of uncertain cases. The final pipeline achieved high F1 scores (exceeding 96%) across all three classes.

Furthermore, we applied the pipeline to a large corpus of PK literature, enhancing the open-source PK paper search tool by enabling the identification of in vivo PK data tables in open-access publications. This work represents a valuable foundation for more efficient, large-scale PK data extraction and integration.

## Supplementary Information

Supplementary Information.

## Data Availability

Data is available from: https://zenodo.org/records/13884895, License: MIT.

## Software availability

Source code is available from: https://github.com/PKPDAI/PKTabClassifier, License: MIT.
