# Supplementary material for: An automated classification pipeline for tables in pharmacokinetic literature
Source: Sci Rep. 2025 Mar 24;15:10071. doi: 10.1038/s41598-025-94778-5 (PMC11933424; doi:10.1038/s41598-025-94778-5)
Supplement: Supplementary file 1 — Supplementary Information. [file 41598_2025_94778_MOESM1_ESM.pdf]

Appendices

A Annotation

A.1 Annotation Interface

A screenshot of the annotation interface can be seen in Figure 1.

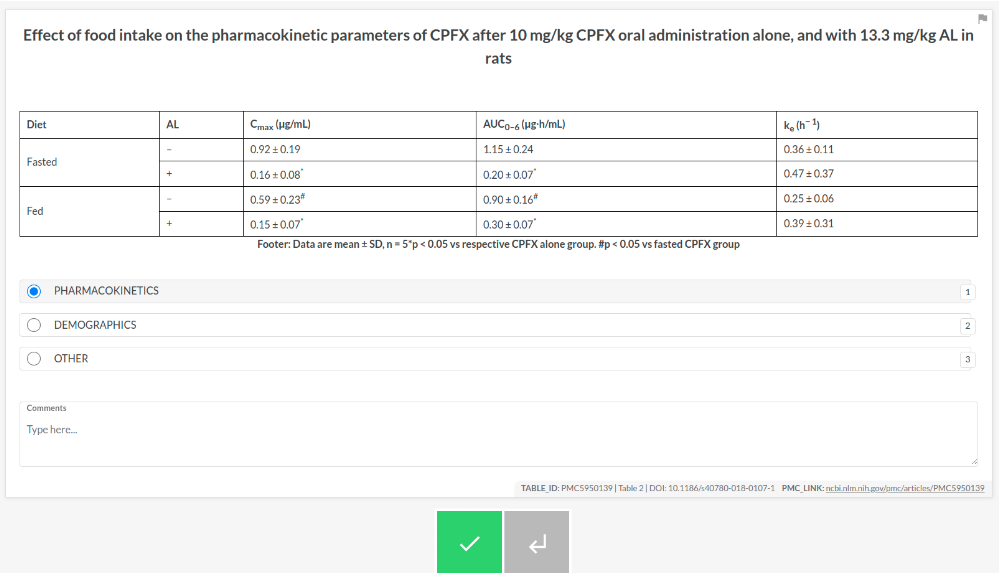

Figure 1: Screenshot of the interface built in Prodigy used to annotate PK tables from scientific literature.

A.2 Annotation Guidelines

The PK table annotation guidelines can be seen below:

ANNOTATION GUIDELINES:
PK TABLE CLASSIFICATION

Project Background

Biomedical Literature contains a wealth of information on Pharmacokinetics (PK), including parameters and covariates, often reported in detail in tables. This data is helpful for preclinical PK predictions and initiating Population PK Models. However, it takes time for PK researchers to extract information from the literature manually. Extracting this data automatically from tables would enable accelerated curation of PK databases for research and drug development. Computationally, extracting data from tables is a complex task, which can be broken down into the following steps: (a) table type classification (classify if the table is relevant and what it contains), (b) recognise the entities within the table cells (e.g., PK parameters, units, numeric values) and link these to a structured knowledge base, and (c) understand the relations between entities in cells of the tables (how rows and columns relate) to extract comprehensive data.

Aim: In this study, we aim to develop a classification pipeline to identify tables containing PK parameter data and tackle step (a) of automated table data extraction in PK.

Method: We sample tables from PubMed OpenAccess reporting PK parameters in their abstract. Then, we ask annotators to label tables with three labels corresponding to whether they report in vivo pharmacokinetic parameters, study population characteristics (demographics) or others.

Task Overview & Annotation Interface

The interface displays a single table, its caption and footer (if one is present) and the annotator must select a label out of three possible choices for the table. Two annotators are assigned to annotate each table; a third annotator checks disagreements and raises these to discuss with the team where necessary.

Please see details of the key functionality of the labelling interface below:

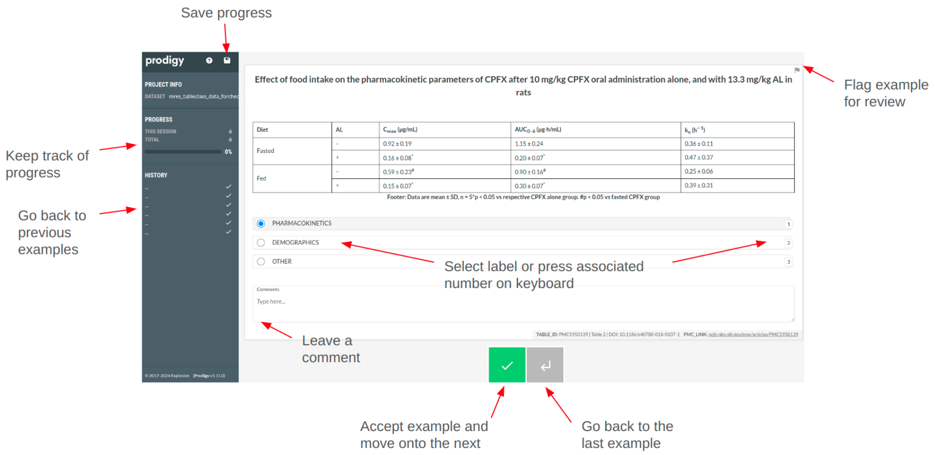

Saving Annotations: If your annotations are saved correctly, you will see a green box in the right-hand corner when you press the floppy disk (in the top left-hand corner). If this box is red, an error occurs on the server, so please raise this immediately.

Viewing Guidelines: You can quickly view these guidelines inside the interface by pressing the question mark in the top left-hand corner.

Further Information: A link to the original paper from which the table is taken, and the table number is provided to allow information to be checked against the paper text if needed.

What if I'm unsure? Please flag the example using the flag icon in the top right-hand corner and leave a comment in the box. The team can then review and discuss it.

Label Definitions

LABEL	DESCRIPTION
**PHARMACOKINETICS**	Select for any table containing newly estimated PK parameters obtained in vivo, for example:<br>• Parameter estimates from non-compartmental analyses (e.g., AUC, Cmax, Tmax).<br>• Parameter estimates from a compartmental analysis (e.g., volume, clearance, and micro and macros rate constants).<br>• Please refer to this article for a detailed ontology of in vivo PK parameters.
**DEMOGRAPHICS**	Select any table reporting:<br>• patient or animal characteristics (demographic) information which does not also report PK parameters.
**OTHER**	Select this for any table **not reporting** newly estimated PK parameters obtained in vivo, for example tables presenting:<br>• Only concentration measurements of administered drug in different body fluids (e.g. plasma, whole blood, CSF etc.) at various time points, with no associated PK parameters stated.<br>• P values of parameters only with no estimates.<br>• Adverse events information.<br>• Chemical parameters from in vitro experiments.<br>• PK parameter estimates quoted from previous studies or public resources.<br>• Pharmacodynamic parameters (e.g. AUC/MIC).<br>• PBPK parameters (e.g. blood flow, volume, tissues).<br>• Creatinine or albumin clearance (as this does not relate to the clearance of an administered compound and is thus considered a covariate in PK).<br>• Stability tests of compounds.<br>• Extraction recovery tests.

Examples

- PHARMACOKINETIC - newly estimated in vivo PK parameters reported:

Pharmacokinetic parameters of paeoniflorin and benzoic acid by oral administration in normal rats and rats pretreated with antibiotics.

Parameters	Units	Normal group		PGF group	
		Paeoniflorin	Benzoic acid	Paeoniflorin	Benzoic acid
Tmax	h	0.458 ± 0.195	0.792 ± 0.188	0.388 ± 0.136	0.597 ± 0.239
t1/2z	h	1.296 ± 0.474	2.555 ± 0.823	2.582 ± 1.614	2.006 ± 0.789
Cmax	μg/L	119.36 ± 54.3	55.58 ± 12.09	133.91 ± 48.55	27.62 ± 2.63
AUC (0-t)	μg/L*h	245.29 ± 72.811	151.24 ± 29.59	296.08 ± 100.14	94.85 ± 19.82
AUC (0-∞)	μg/L*h	246.07 ± 73.073	161.60 ± 40.10	312.15 ± 123.07	97.18 ± 20.23

Footer: Each value represents mean ± SD.

- OTHER - only PD parameters:

Fosfomycin dosing regimens, based on bloodstream PK data, applied in the PK/PD study in the experimental UTI modela

Dose (mg/mouse)	Dosing interval (h)	No. of doses per 72-h treatment interval	Total dose (mg)	Value of the following PK/PD index:		
				$T_{>MIC}$ (%)	AUC/MIC (h^{-1})	C_{max}/MIC
30	72	1	30	9	607	750
15	36	2	30	14	727	468
7.5	72	1	7.5	4	212	281
1.88	6	12	22.56	42	635	78
0.47	6	12	5.64	30	158	22
0.47	12	6	2.82	15	79	22

Footer: aTreatment was initiated at 24 h postinfection, and the treatment period was 72 h.

- OTHER - only p values for PK parameters, no parameter estimates:

Statistic analysis of the pharmacokinetic parameters

Group	P-value for $t_{1/2}$	P-value for F_{0-t}	P-value for T_{max}
EDR suspension (172 μM/kg) vs NEF (46 μM/kg)	0.0252	<0.0001	0.9979
EDR suspension (172 μM/kg) vs NEF (138 μM/kg)	0.0005	<0.0001	0.9958
EDR suspension (172 μM/kg) vs NEF (414 μM/kg)	<0.0001	<0.0001	0.9914
NEF (46 μM/kg) vs NEF (138 μM/kg)	0.3147	0.0076	>0.9999
NEF (46 μM/kg) vs NEF (414 μM/kg)	0.0714	0.0448	0.9995
NEF (138 μM/kg) vs NEF (414 μM/kg)	0.8305	0.8460	>0.9999

Footer: Notes: t1/2, half-life; Tmax, peak time.Abbreviations: EDR, edaravone; NEF, novel EDR formulation.

- OTHER - only demographic information from previous studies, no newly reported data:

Published studies on the pharmacokinetics of inhaled salbutamol (updated on the 31st July 2014)

	Physical activity	Asthmatic status	Salbutamol inhaled dose	Rest or exercise	Exercise intensity	Sample size
Anderson1998 [26]	Untrained	Healthy	1 × 180 µg	Rest		10
Berges2000 [11]	Trained	Asthmatic	1 × 200 µg	Rest		15
		Healthy	4 × 400 µg			17
Pichon2006 [27]	Trained	Healthy	3 × 200 µg	Rest		10
Sporer2008 (1) [28]	Trained	Healthy	1 × 200 µg<br>and 1 × 400 µg<br>and 1 × 800 µg	Exercise (and Rest)	Cycling time	30
Sporer2008 (2) [29]	Untrained	Healthy	1 × 200 µg	Rest		8
Elers2010 [30]	Untrained	Asthmatic	1 × 200 µg	Rest		10
Elers2011 [31]	Untrained	Asthmatic	4 × 400 µg	Rest		10
		Healthy				10
Elers2012 [19]	Trained	Asthmatic<br>Healthy	1 × 800 µg	Rest		10
Dickinson2014 [9]	Trained	Healthy	1 × 800 µg<br>and 1 × 1600 µg	Exercise (and Rest)	Not specified	32
Theoretical "Link-field study"	Trained	Asthmatic	Preventive dose:<br>3 × 200 µg	Exercise (and Rest)	Exercise	Powerful

Footer: Bold data identify conditions that we consider as optimal based on a rigorous clinical and scientific approach

- PHARMACOKINETIC - although it includes in vitro estimates, in vico PK parameters are also estimated:

Pharmacokinetic parameters of CUMYL-PICA and 5F-CUMYL-PICA incubated in rat and human liver microsomes in vitro and in rat plasma in vivo

**Pharmacokinetic parameter**	**CUMYL-PICA**	**5F-CUMYL-PICA**
Rat liver microsomes		
Half-life (min)	2.24	1.19
$CL_{int,micr}$ (mL/min/mg)	0.31	0.58
CL_{int} (mL/min/kg body wt.)	556.88	1048.24
CL_H (mL/min/kg body wt.)	50.22	52.44
ER	0.91	0.95
Human liver microsomes		
Half-life (min)	5.92	1.77
$CL_{int,micr}$ (mL/min/mg)	0.12	0.39
CL_{int} (mL/min/kg body wt.)	135.46	453.05
CL_H (mL/min/kg body wt.)	17.43	19.15
ER	0.87	0.96
Rat plasma		
Half-life (h)	7.26	12.00
CL/F (mL/min/kg body wt.)	43.31	147.88
C_{max} (ng/mL)	130.50	65.25
T_{max} (h)	0.50	0.50
AUC 0–24 h (h ng/mL)	1086.57	581.78
AUC 0–∞ (h ng/mL)	1214.85	843.28

Footer: AUC area under the curve, CL/F observed apparent clearance, CL H estimated hepatic clearance, CL int estimated intrinsic clearance, CL int, micr intrinsic microsome clearance, C max mean maximum observed concentration, ER extraction ratio, T max mean time of C max

B Classification Pipeline

B.1 Chain-of-Thought Prompt

The prompt used for classification by LLM is as follows:

You are a highly intelligent and accurate scientific table classifier with reasoning capabilities. You will receive a table and its caption from the scientific literature. Your job is to classify this into one of three possible classes: Pharmacokinetic, Demographic, Other.

Please answer the following questions to determine the output class. Questions:

Q1. Pharmacokinetic Results: Does the table reports parameters from a Pharmacokinetic (PK) analysis, obtained in vivo? Please note this excludes the following:

- PK parameter estimates quoted from previous studies or public resources.
- Pharmacodynamic parameters (e.g. AUC/MIC).
- PBPK parameters (e.g. blood flow, volume, tissues).
- Only concentration measurements of administered drug (e.g. in plasma, whole blood, CSF etc.) at various time points, with no associated PK parameters stated.
- P values of parameters only with no estimates.
- Parameters from in vitro experiments.
- Creatinine or albumin clearance only.
- Stability tests of compounds.
- Extraction recovery tests.

If the answer to Q1 is yes, set the final answer to Pharmacokinetic. Otherwise, go to Q2.

Q2. Does the table report demographic information from a study population (of either humans or animals). Please note this excludes adverse events information. If the answer to Q2 is yes, set the final answer to Demographic. Otherwise set the final answer to Other.

Caption: caption Table: table

Please return only the final answer in the format "Answer": "final answer".

B.2 Feature Token Lengths

The token lengths of different table features are shown in Table 1.

Table Field/s	Token Lengths	# > 512 tokens (%)
Table (serialized rows)	288(24, 6825)	604 (23)
Caption + Table	312(37, 6870)	661 (25)

Table 1: Token lengths of long table features shown as median (range) for the full dataset using the bert-based-uncased tokenizer.

B.3 Final XGBoost Model Hyperparameters

The final hyperparameters used in the XGBoost model following hyperparameter tuning can be seen in Table 2.

C Establishing the Confidence Threshold

Histograms of confidence scores on the validation and test sets were generated to observe the confidence score distributions.

For each confidence threshold (T) between 0.5 and 0.95 in 0.05 intervals, the F1-score was plotted for all samples with confidences falling under the confidence threshold. The percentage of the dataset under the threshold was also

**Hyperparameter**	**Value**
learning rate	0.1
max depth	2
min child weight	1
gamma	0
subsample	0.5
colsample bytree	0.3
n estimators	132 (from early stopping)

Table 2: Tuned XGBoost hyperparameters used in the final best classification pipeline.

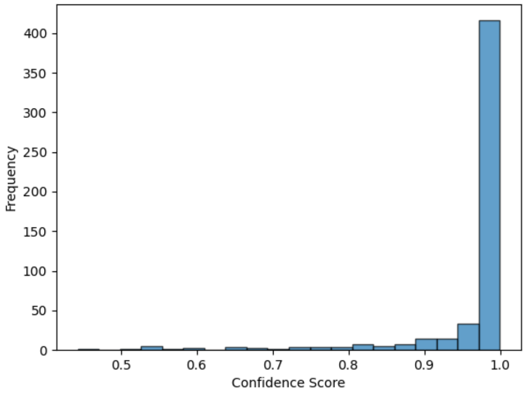

(a) Validation

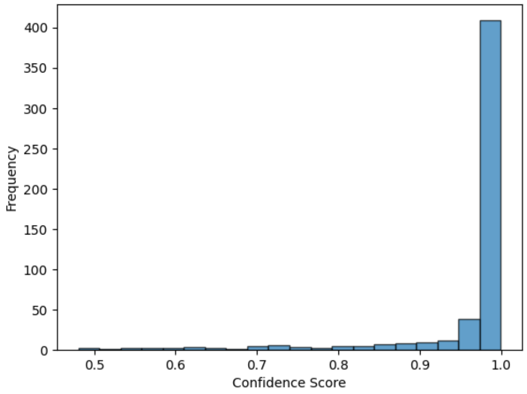

(b) Test

Figure 2: Histograms of confidence scores for validation and test sets.

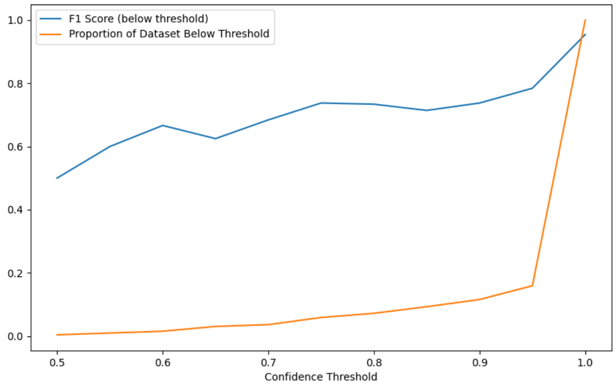

Figure 3: Plot of F1 score and dataset percentage falling under various confidence thresholds on the validation set.

plotted. T values were selected to minimise the number of samples under the threshold but where the model was underperforming.

A histogram of confidence scores was also generated on the full dataset during the large-scale application. This was to validate that the distribution closely follows what was observed on the validation and test datasets, so the confidence threshold chosen for those is applicable here.

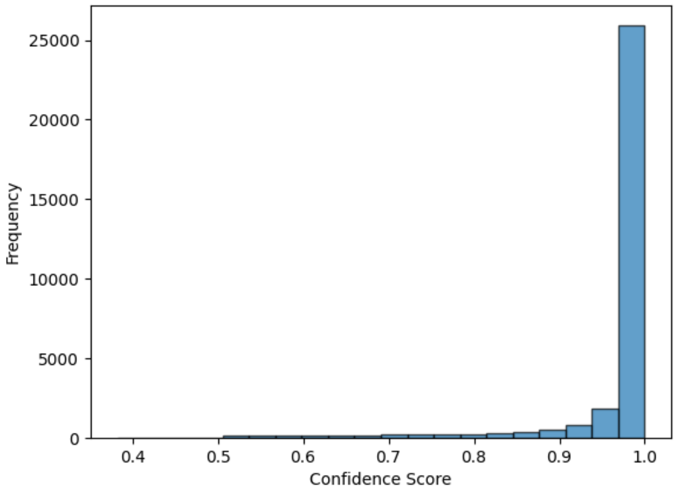

Figure 4: Histogram of confidence scores on the large scale application dataset.
